# STAT3 palmitoylation initiates a positive feedback loop that promotes the malignancy of hepatocellular carcinoma cells in mice

**DOI:** 10.1126/scisignal.add2282

**Published:** 2023-12-05

**Authors:** Yi Jiang, Yuejie Xu, Chengliang Zhu, Guifang Xu, Lei Xu, Zijian Rao, Lixing Zhou, Ping Jiang, Sara Malik, Jingyuan Fang, Hening Lin, Mingming Zhang

**Affiliations:** 1 Division of Gastroenterology and Hepatology; Shanghai Institute of Digestive Disease; NHC Key Laboratory of Digestive Diseases; State Key Laboratory for Oncogenes and Related Genes; Renji Hospital, School of Medicine, Shanghai Jiao Tong University, Shanghai 200001, China; 2 Department of Gastroenterology, Nanjing Drum Tower Hospital, the Affiliated Hospital of Nanjing University Medical School, Nanjing University, Nanjing 210008, China; 3 Department of Traditional Chinese Medicine, Nanjing Drum Tower Hospital Clinical College of Traditional Chinese and Western Medicine, Nanjing University of Chinese Medicine, Nanjing 210046, China; 4 Institute of Pharmacology and Toxicology, Zhejiang Province Key Laboratory of Anti-Cancer Drug Research, College of Pharmaceutical Sciences, Zhejiang University, Hangzhou 310058, China; 5 Center for Drug Safety Evaluation and Research of Zhejiang University, Zhejiang University, Hangzhou 310058, China; 6 The Center of Gerontology and Geriatrics, West China Hospital, Sichuan University, Chengdu, 610041, China; 7 Northwestern University Feinberg School of Medicine, Chicago, 60611, IL, United States; 8 Howard Hughes Medical Institute; Department of Chemistry and Chemical Biology, Department of Molecular Biology and Genetics, Cornell University, Ithaca, NY 14853, United States

**Keywords:** DHHC7, STAT3, S-palmitoylation, HIF1α, hepatocellular carcinoma

## Abstract

Constitutive activation of the transcription factor STAT3 (signal transducer and activator of transcription 3) contributes to the malignancy of many cancers such as hepatocellular carcinoma (HCC) and is associated with poor prognosis. STAT3 activity is increased by the reversible palmitoylation of Cys^108^ by the palmitoyltransferase DHHC7 (encoded by *ZDHHC7*). Here, we investigated the consequences of S-palmitoylation of STAT3 in HCC. Increased *ZDHHC7* abundance in HCC cases was associated with poor prognosis, as revealed by bioinformatics analysis of patient data. In HepG2 cells in vitro, DHHC7-mediated palmitoylation enhanced the expression of STAT3 target genes, including *HIF1A*, which encodes the hypoxia-inducible transcription factor HIF-1α. Inhibiting DHHC7 decreased the S-palmitoylation of STAT3 and decreased HIF-1α abundance. Furthermore, stabilization of HIF-1α by cyclin-dependent kinase 5 (CDK5) enabled it to promote the expression of *ZDHHC7*, which generated a positive feedback loop between DHHC7, STAT3, and HIF-1α. Perturbing this loop reduced the growth of HCC cells in vivo. Moreover, DHHC7, STAT3, and HIF-1α were all abundant in human HCC tissues. Our study identifies a pathway connecting these proteins that is initiated by S-palmitoylation, which may be broadly applicable to understanding the role of this modification in cancer.

## One-sentence summary

Liver tumor growth in mice is exacerbated by a feed forward loop triggered by the palmitoylation of STAT3.

## Introduction

Hepatocellular carcinoma (HCC) is a highly aggressive and metastatic neoplasm with poor prognosis, the incidence of HCC and HCC-related deaths have increased over the last several decades, treatment options for advanced HCC are limited(2). Although multi-tyrosine kinase inhibitors (MTKIs) like sorafenib and lenvatinib are approved for advanced HCC with a 5-year survival rate of only about 18%(3) and a new therapeutic strategy combining atezolizumab with bevacizumab resulted in a better 1-year survival rate compared to sorafenib(4), new biomarkers and therapies for HCC are still needed.

Signal transducer and activator of transcription 3 (STAT3) is a transcription factor that is important for various malignant and inflammatory processes(1, 3, 5, 6). Under specific cytokine stimulation, activated STAT3 promotes the expression of downstream target genes, such as *HIF1A* (hypoxia inducible factor-1 alpha) and *MCL1* (myeloid cell leukemia-1), which are involved in tumorigenesis and proliferation(3, 7–9). Constitutive activation of STAT3 contributes to the malignancy in the majority of cancers such as HCC and is associated with poor prognosis(3, 10). As a downstream target gene of STAT3, MCL1 artificially co-overexpressed with c-MYC in liver induces in situ tumorigenesis in many different mouse strains(9). Given the constitutive activation and critical oncogenic roles of STAT3 in cancers, STAT3 has become an emerging therapeutic target.

Cysteine palmitoylation (S-palmitoylation) is a post-translational modification that impels proteins to migrate to membrane and promotes the malignancy of many cancer cells(11–14). S-palmitoylation is catalyzed by 23 mammalian palmitoyltransferases (DHHCs), which share the Asp-His-His-Cys sequence motif(11), and is removed by acyl protein thioesterases (APT and ABHD family members)(15). Although both DHHCs and APTs are known to work on numerous proteins(12), the physiological function of S-palmitoylation in HCC has not been reported. STAT3 is palmitoylated on Cys108 by DHHC7 (gene name *ZDHHC7*) and reversed by APT2 (also called LYPLA2)(1). A STAT3 palmitoylation-depalmitoylation cycle promotes Th17 cell differentiation and inflammatory bowel disease (IBD)(1). However, the involvement of STAT3 S-palmitoylation in cancer and the regulation of S-palmitoylation has not yet been reported.

*HIF1A* is a target gene of STAT3 and important in hypoxia-responsive pathway(8). In HCC, HIF1α is constitutively phosphorylated at Ser687 and stabilized by cyclin dependent kinase 5 (CDK5)(16). Accumulated HIF1α heterodimerizes with HIF1β (Arylhydrocarbon receptor nuclear translocator, ARNT), binds to core hypoxia-response element (HRE, 5′-(A/G)CGTG-3′), and promotes the transcription of the target genes(16). Because both CDK5 and HIF1α are positively associated with the poor survival of HCC patients and blockage of either CDK5 or HIF1α is a promising approach for HCC treatment(16, 17). we investigated the effect of STAT3 S-palmitoylation on HIF1α in HCC.

Here, we report that S-palmitoylation of STAT3 stimulates the malignancy of human HCC cell lines, HepG2 cells and PLC/PRF/5 cells, and contributes to the poor prognosis of HCC patients. S-palmitoylation by DHHC7 enhances STAT3 transcriptional activity and promotes the transcription of target genes such as *HIF1A* and *MCL1*. HIF1α in turn promotes *ZDHHC7* gene expression. This creates a positive-feedback loop that contributes to the malignancy of HCC. Perturbing this positive feedback loop by targeting DHHC7 pharmacologically or genetically relieves the malignancy of HCC in vitro and in vivo. Our findings reveal a potential new therapeutic strategy for treating HCC and may be broadly applicable to other cancers and inflammatory diseases.

## Results

### DHHC7 is associated with the malignancy of HCC.

**Fig. 1.**
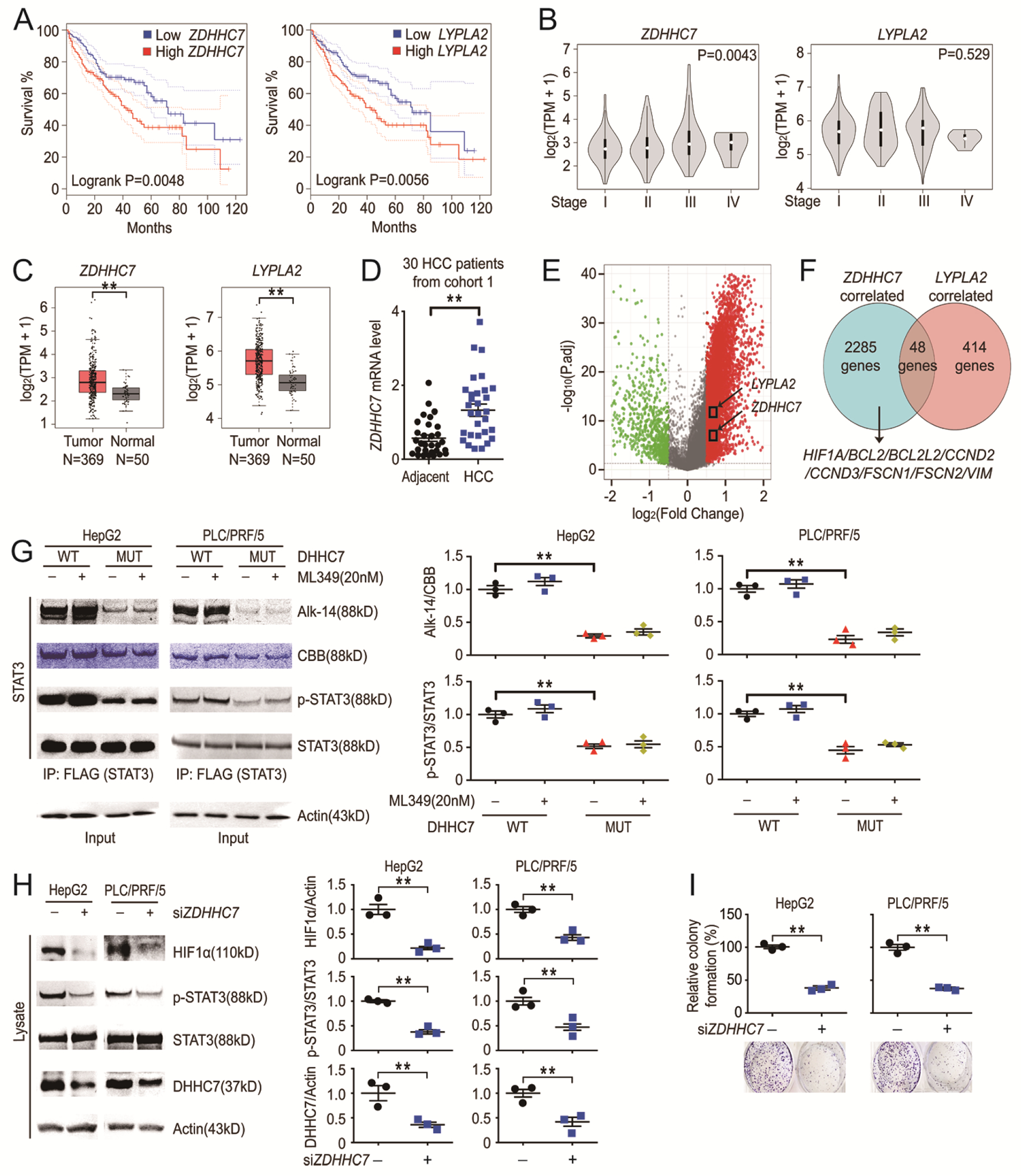
DHHC7 contributes the malignancy of HCC. (**A** and **B**) Expression of *ZDHHC7* and *LYPLA2* genes in HCC (N = 369) samples from TCGA data were visualized using Gepia (http://gepia.cancer-pku.cn/). (A) Kaplan-Meier curves of *ZDHHC7* and *LYPLA2* genes were performed in Gepia using HCC samples. (B) Expression of *ZDHHC7* and *LYPLA2* genes in different stages of HCC patients (N = 369) were performed in Gepia. (**C**) Differentially expressed *ZDHHC7* and *LYPLA2* genes in HCC (N = 369) and normal liver (N = 50) samples from TCGA data were visualized using Gepia (http://gepia.cancer-pku.cn/). TPM, Transcripts Per Million. (**D**) Human cDNA reverse transcripts from 30 patients with HCC (Cohort 1), and *ZDHHC7* were analyzed using rtPCR. N = 30 HCC patients. (**E**) Volcano plot of differentially expressed genes (DEGs) in HCC (N = 369) and normal liver (N = 50) samples from TCGA data was visualized. An over 1.41-fold increase and decrease in DEGs in HCC are shown in red and green respectively. (**F**) Venn diagram of both *ZDHHC7* and *LYPLA2* correlated genes was performed using HCC (N = 369) samples from TCGA data (the absolute Spearman’s correlation R value is above 0.5). (**G**) HCC cells were transfected with indicated Flag-STAT3,DHHC7 WT, or catalytic mutant counterparts. The cells were labeled with or without Alk14. STAT3 was pulled down, followed by in-gel fluorescence or western blot analyses (left) and quantified (right) as indicated. CBB was the coomassie brilliant blue staining of STAT3. N = 3 biological replicates over 3 independent experiments. (**H**) Western blots of *ZDHHC7* knockdown HCC and control cells (left) and quantified (right). N = 3 biological replicates over 3 independent experiments. (**I**) Colony formation assay of *ZDHHC7* knockdown HCC cells and control cells was performed and colony numbers were counted and normalized (top) and quantified (bottom) as indicated. N = 3 biological replicates over 3 independent experiments. Data represent the Mean ± SEM. ** p < 0.01, by Two-tailed unpaired student’s t-test.

S-palmitoylation is catalyzed by palmitoyltransferases (DHHC family members)(11), and removed by acyl protein thioesterases (APT and ABHD family members)(15). Although S-palmitoylation promotes the malignancy of many cancer cells(11–14), the association between S-palmitoylation and HCC has not been reported. We screened for all DHHCs, APTs and ABHDs family members using mRNA expression data of 369 HCC samples and 50 normal samples from Gepia (Gene Expression Profiling Interactive Analysis from http://gepia.cancer-pku.cn/), which is a web-based tool to deliver fast and customizable functionalities based on TCGA (The Cancer Genome Atlas) data(18). We found that *ZDHHC7* was most relevant to malignancy of HCC among palmitoyltransferases according to expression level, prognosis and tumor stage. *LYPLA2* is the only depalmitoacylase that is associated with both tumor stage and prognosis of HCC. (Fig. 1, A to C and fig. S1 and fig. S2 and fig. S3A). Moreover, reverse transcripts of cDNA from 30 HCC patients (cohort 1) (Table S1) were analyzed and *ZDHHC7* was more abundantly expressed in HCC tissues compared to the adjacent counterparts (Fig. 1D).

Given that the palmitoylation-depalmitoylation cycle induced by DHHC7 and APT2 is important for STAT3 activation(1) and the expression of downstream target genes such as *HIF1A, CCND1* (G1/S-specific cyclin-D1), *BCL2L1* (Bcl-2-like protein 1), *VIM* (Vimentin), and *ICAM1* (Intercellular adhesion molecule 1)(3, 7, 8) (fig. S3B), we further inquired how S-palmitoylation-related genes are regulated in HCC. We analyzed the differentially expressed genes (DEGs) in HCC using TCGA data. From a total of 19463 mRNA expression values from 375 HCC samples and 50 normal samples, we identified the differentially expressed genes including *ZDHHC7* and *LYPLA2* (Fig. 1E). Bioinformatics analyses showed that many STAT3 downstream target genes were positively correlated with *ZDHHC7* but not *LYPLA2* (Fig. 1F). These data indicate that DHHC7 is a major contributor to the S-palmitoylation of STAT3 and associated with the malignancy of HCC.

### DHHC7 promotes STAT3 activity and cell proliferation in HCC.

Next, different HCC cell lines were used to evaluate how the activity of STAT3 was regulated and we found that both S-palmitoylation (Fig. 1G) and phosphorylation (Fig. 1, G and H) of STAT3 could be induced by DHHC7 WT while APT2 inhibition with ML349 (a specific APT2 inhibitor)(1) only caused minor perturbation to STAT3 phosphorylation (Fig. 1G).

We further visualized the ability of different DHHCs and APTs in promoting the proliferation of HCC cells and found only DHHC7 increased the cell colony formation significantly (fig. S3C and S3D). Since the sequences of *ZDHHC3/20* were close to *ZDHHC7* (19) and *ZDHHC3/20* were more abundantly expressed in HCC compared to normal tissues (fig. S3A), we further used *ZDHHC3/20* as control genes to evaluate the function of *ZDHHC7* in cellular colony formation. We found that *ZDHHC7* knockdown significantly inhibited the proliferation of different HCC cell lines (Fig. 1I) while *ZDHHC3/20* silencing in HCC cells had no significant effect on proliferation (fig. S3E). Together, the results support that DHHC7-catalyzed S-palmitoylation promotes the cell proliferation of HCC.

### CDK5 and HIF1α are upstream stimulators of *ZDHHC7* gene expression.

**Fig. 2.**
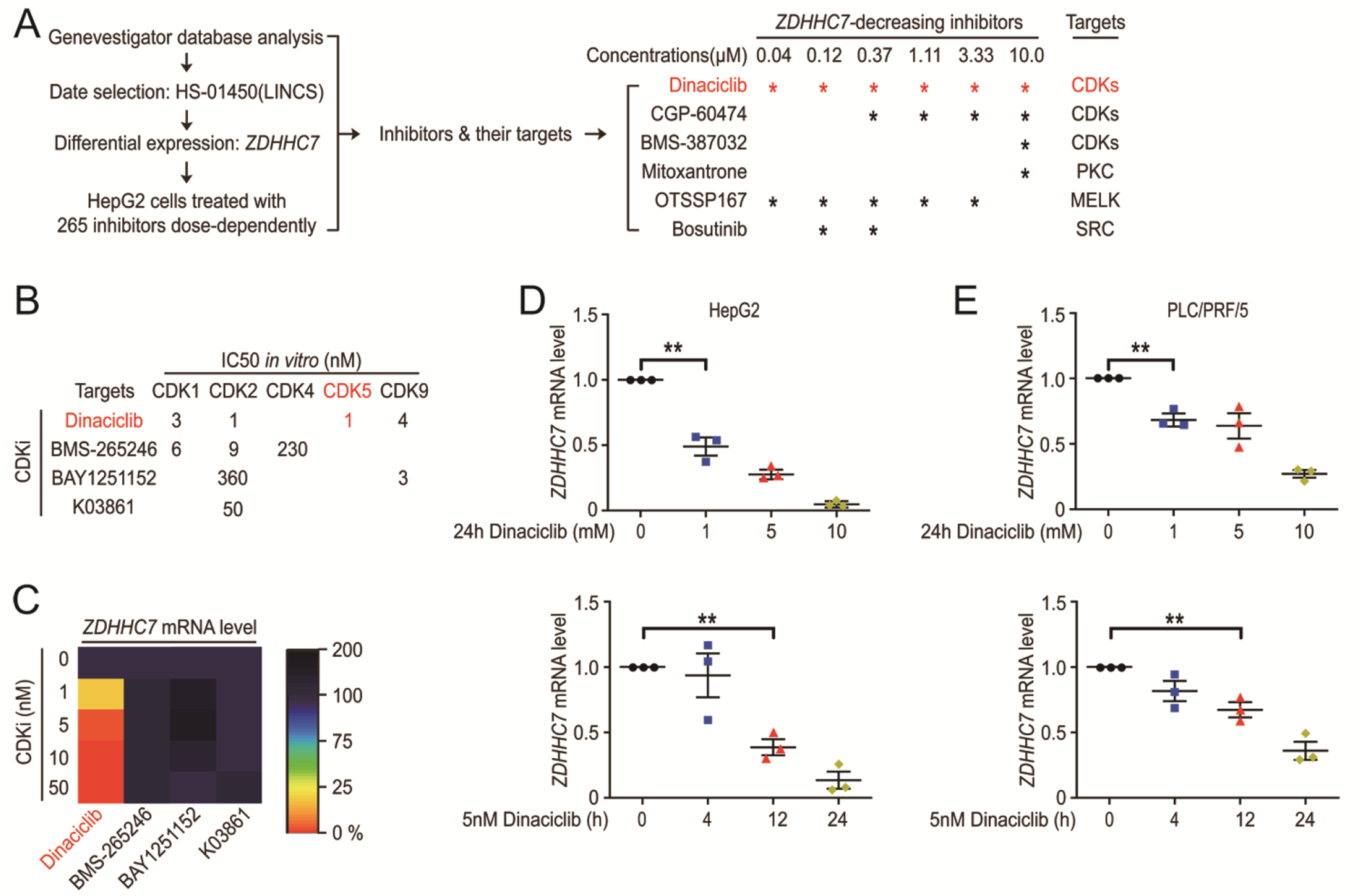
CDK5 and HIF1α are the upstream stimulators of *ZDHHC7* gene expression. (**A**) Scheme showing the identification of small molecules that down regulate the *ZDHHC7* gene expression using Genevestigator (left). All of the inhibitors and their targets and concentrations used are listed (right) and * indicates decreased ZDHHC7 expression by the inhibitor at the concentration used. (**B**) CDK inhibitors and their in vitro IC50 values. (**C**) HepG2 cells were treated with different CDK inhibitors and total mRNA measured by rtPCR. Relative change compared to control is shown. N = 3 biological replicates over 3 independent experiments. (**D** and **E**) HepG2 cells and PLC/PRF/5 cells were treated with dinaciclib. Total mRNA was analyzed by rtPCR, and the relative change of *ZDHHC7* compared to the control was shown. N = 3 biological replicates over 3 independent experiments. ** p < 0.01, by Ordinary one-way ANOVA.

Given that DHHC7 is the major promotor for STAT3 activation in HCC cells, we screened for the upstream regulator of DHHC7 using the bioinformatics tool Genevestigator, which contains previously published microarray mRNA expression data (20, 21). In the data set HS-01450 (Library of Integrated Network-Based Cellular Signatures, LINCS), which include microarray data from HCC cell line (HepG2) treated with 265 inhibitors at several doses (Fig. 2A), we found that many inhibitors significantly reduced *ZDHHC7* gene expression at various concentrations and most of the inhibitors were CDK inhibitors (dinaciclib, CGP-60474 and BMS-387032) (Fig. 2A). In addition, dinaciclib was the only inhibitor that was effective at all concentrations (Fig. 2A). Dinaciclib has strong inhibition on CDK1/2/5/9, and is the enhancer for chemotherapy response in HCC(22). To find which CDK is associated with *ZDHHC7* gene expression, we purchased inhibitors with different CDK specificities (Dinaciclib for CDK1/2/5/9, BMS-265246 for CDK1/2, BAY1251152 for CDK9 and K03861 for CDK2) (Fig. 2B). Dinaciclib, as the only CDK5 inhibitor among these commercially purchased inhibitors, reduced the *ZDHHC7* expression at a concentration as low as 1 nM while other CDK inhibitors had little effect (Fig. 2, C to E). Thus, CDK5 appears to be an upstream regulator of *ZDHHC7* expression.

**Fig. 3.**
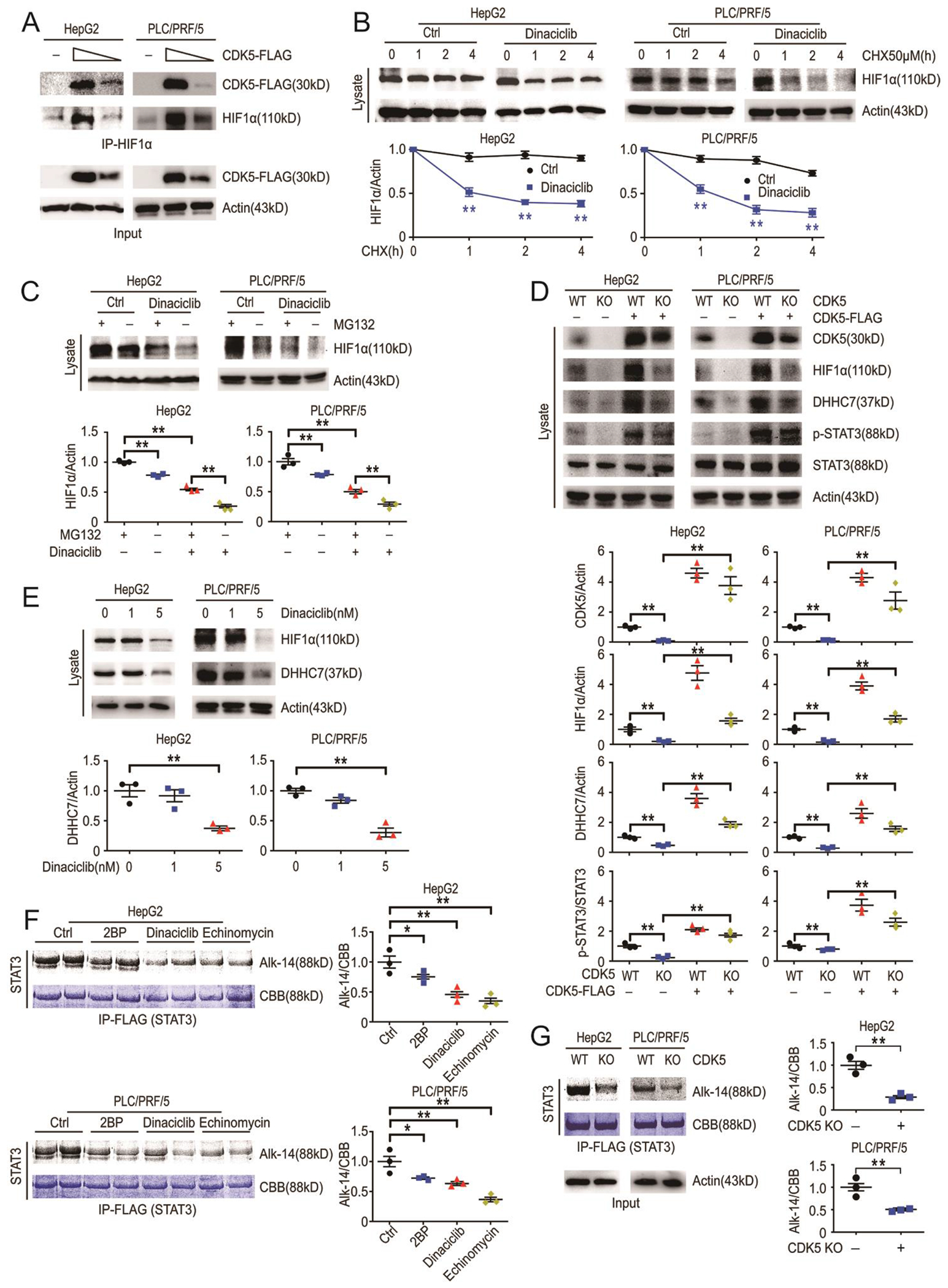
CDK5 promotes *ZDHHC7* gene expression by stabilizing HIF1α. (**A**) HCC cells were transfected with control or Flag-CDK5 plasmids. Endogenous HIF1α was pulled down and western blot was performed. N = 3 biological replicates over 3 independent experiments. (**B**) HCC cells treated with CHX as indicated, with or without dinaciclib. Endogenous HIF1α was measured by western blot (left) and quantified (right). N = 3 biological replicates over 3 independent experiments. (**C**) HCC cells were treated with dinaciclib, with or without MG132. Endogenous protein was measured by western blot (top) and quantified (bottom). N = 3 biological replicates over 3 independent experiments. (**D**) WT and CDK5 knockout (KO) cells with or without CDK5 overexpression (OE) were treated with MG132. Endogenous protein targets were visualized by western blot analyses (top) and quantified (bottom) as indicated. N = 3 biological replicates over 3 independent experiments. (**E**) HCC cells were treated with indicated concentrations of dinaciclib. Endogenous protein was measured by western blot (top) and quantified (bottom). N = 3 biological replicates over 3 independent experiments. (**F** and **G**) WT and CDK5 KO HCC cells were transfected with Flag-STAT3 WT and labeled with Alk14. After treatment with inhibitors (2BP, dinaciclib or echinomycin), STAT3 was pulled down and palmitoylation of STAT3 was measured by in-gel fluorescence including coomassie brilliant blue (CBB) staining of STAT3 (left) and quantified (right). N = 3 biological replicates over 3 independent experiments. Data represent the Mean ± SEM. * p < 0.05, ** p < 0.01, by Two-tailed unpaired student’s t-test.

HIF1α is constitutively stabilized by CDK5(16) and moreover, both CDK5 and HIF1α are well established cancer promoters in multiple tumors, including HCC(8, 16). Thus, we next evaluated whether CDK5 induced HIF1α stabilization promotes *ZDHHC7* gene expression in HCC cell lines. We found that CDK5 bound to endogenous HIF1α in a dose-dependent manner (Fig. 3A), and that treatment with the CDK5 inhibitor, dinaciclib, induced a significant destabilization of HIF1α (Fig. 3, B and C). Moreover, we further evaluated the abundance of HIF1α in CDK5 knockout and overexpressed HCC cells and found that CDK5 significantly increased the abundance of HIF1α (Fig. 3D).

Both chemical and biological inhibition of CDK5 significantly decreased DHHC7 abundance as well as the amount of STAT3 S-palmitoylation (Fig. 3, E to G). Moreover, we visualized STAT3 S-palmitoylation after these upstream stimulators were blocked and found that DHHC7, CDK5 and HIF1α inhibition significantly reduced STAT3 S-palmitoylation (Fig. 3, F and G). Both dinaciclib and echinomycin (an inhibitor of HIF1α that blocking its DNA-binding activity(23), Synonyms: Quinomycin A; NSC-13502) reduced STAT3 S-palmitoylation at a concentration as low as 1 nM (Fig. 3F). Thus, CDK5-induced HIF1α stabilization is important for *ZDHHC7* gene expression and STAT3 S-palmitoylation.

### HIF1α is a transcription factor of the *ZDHHC7* gene and is involved in a regulation cycle with DHHC7 to promote STAT3 signaling.

**Fig. 4.**
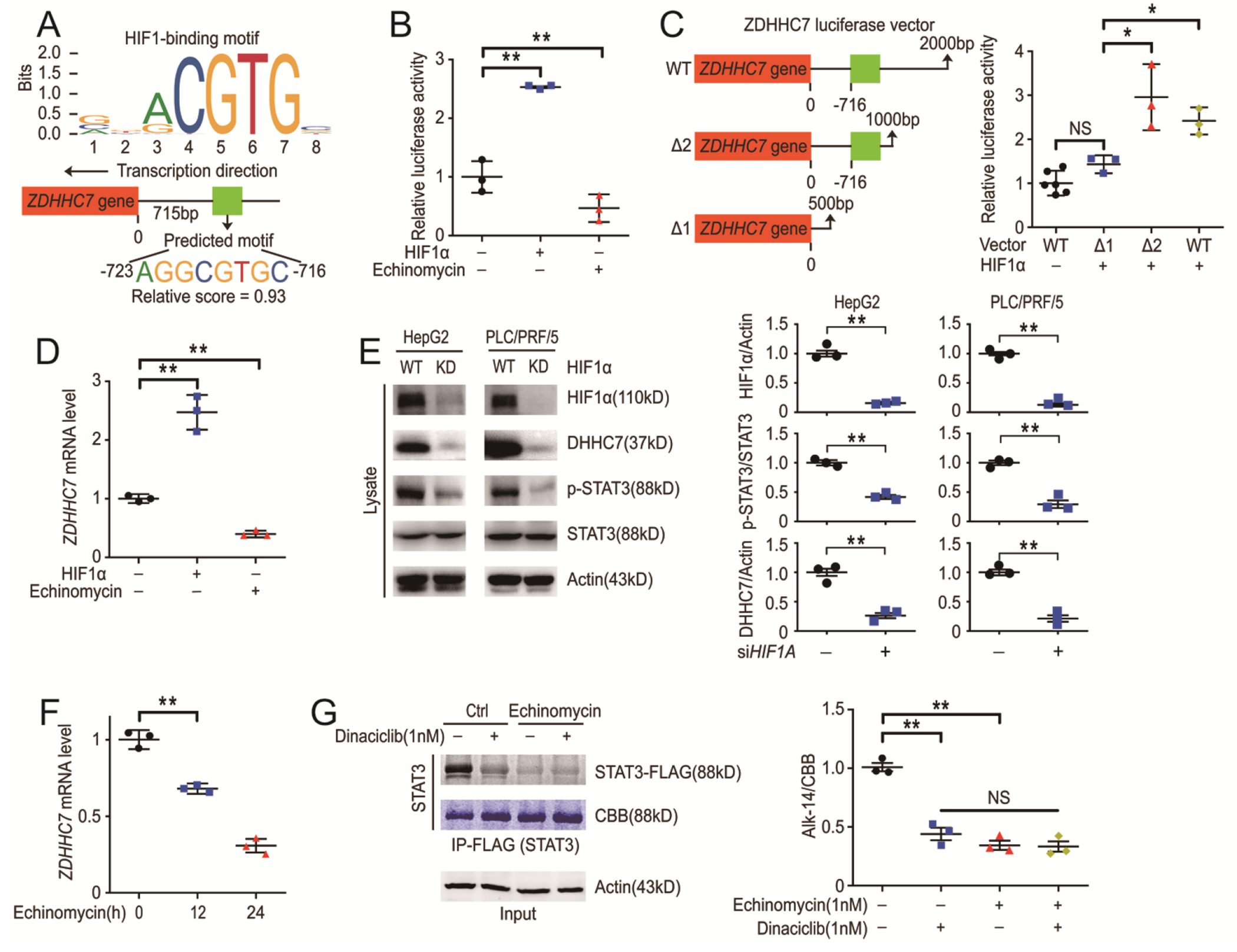
HIF1α is a transcription factor of the *ZDHHC7* gene. (**A**) The core hypoxia-response element (HRE) recognized by HIF1 and the sequence of the predicted HIF1 binding site in the promoter region of *ZDHHC7*. (**B**) Relative luciferase activity was analyzed after *ZDHHC7* reporter plasmids were cotransfected with HIF1α or echinomycin treatment as indicated in 293T cells. N = 3 biological replicates over 3 independent experiments. (**C**) *ZDHHC7* reporter plasmids were mutated as indicated (Δ1 and Δ2) and the relative luciferase activity was analyzed after plasmids were transfected with or without HIF1α as indicated in 293T cells. N = 6/3/3/3 biological replicates over 3 independent experiments. (**D**) HepG2 cells were transfected with HIF1α plasmid or treated with echinomycin . *ZDHHC7* mRNA was analyzed by rtPCR. N = 3 biological replicates over 3 independent experiments. (**E**) Control and HIF1α knockdown HCC cells were harvested and endogenous protein was measured by western blot (left) and quantified (right). N = 3 biological replicates over 3 independent experiments. (**F**) HepG2 cells were treated with echinomycin as indicated. Cells were harvested, and *ZDHHC7* mRNA was analyzed by rtPCR. N = 3 biological replicates over 3 independent experiments. (**G**) 293T cells were transfected with Flag-STAT3 WT and labeled with Alk14. After treating with the indicated inhibitor, STAT3 was pulled down and the palmitoylation of STAT3 was visualized by in-gel fluorescence (left) and quantified (right). CBB was the coomassie brilliant blue staining of STAT3. N = 3 biological replicates over 3 independent experiments. Data represent the Mean ± SEM. * p < 0.05, ** p < 0.01, NS not significant, by Two-tailed unpaired student’s t-test.

HIF1 binds to core hypoxia-response elements (HRE, 5′-(A/G)CGTG-3′) (Fig. 4A) to promote the transcription of target genes(16). We did bioinformatics analyses using JASPAR (a open-access database of curated and non-redundant transcription factor binding profiles), which indicated that HIF1α could be the transcription factor directly regulating the expression of *ZDHHC7*. A 5′-AGGCGTGC-3′ motif in the promoter region of the *ZDHHC7* gene is predicted to the HIF1 binding site (Fig. 4A). Using a luciferase reporter assays with the HIF1 binding site from *ZDHHC7* promoter, we found that HIF1α overexpression significantly increased while echinomycin decreased the transcription of luciferase reporter (Fig. 4B and fig. S3F). The 5′-AGGCGTGC-3′ motif is a HIF1 binding site in the promoter region of *ZDHHC7* gene as when this motif is deleted in the luciferase reporter assay, the expression of luciferase could not be promoted by HIF1α (Fig. 4C). We further inhibited DNA-binding activity of HIF1α with echinomycin in HepG2 cells and found that *ZDHHC7* expression, as well as STAT3 S-palmitoylation, were significantly reduced compared to the control cells (Fig. 4, D to G). Moreover, CDK5 inhibitor treatment was unable to further reduce STAT3 S-palmitoylation in the presence of an HIF1α inhibitor (Fig. 4G) and HIF1α knock-down inhibited both DHHC7 and p-STAT3 (Fig. 4E), which suggests that HIF1α is downstream of CDK5 and that it directly induces *ZDHHC7* gene expression. Given that HIF1α is a transcription factor of the *ZDHHC7* gene, DHHC7-palmitoylates and activates STAT3, and STAT3 promotes *HIF1A* gene expression, our work suggested a positive feedback loop formed by STAT3, HIF1α, and DHHC7.

### STAT3-HIF1α-DHHC7 loop is a therapeutic target of hepatic carcinoma cells.

**Fig. 5.**
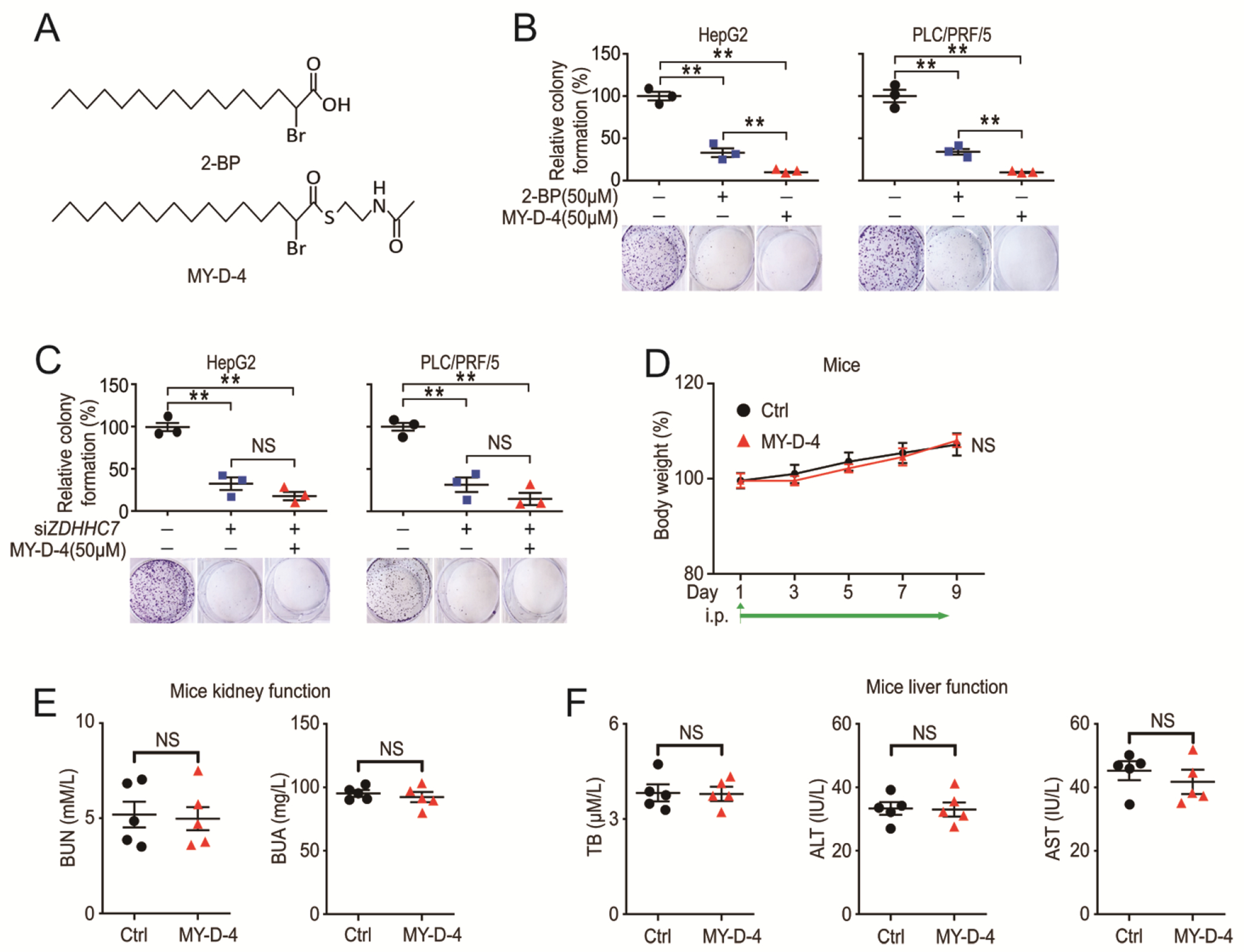
The S-palmitoylation inhibitor MY-D-4 inhibits HCC cell growth. (**A**) Chemical structures of 2-BP and MY-D-4. (**B**) Colony formation assay of HepG2 cell lines and PLC/PRF/5 cell lines treated with inhibitors or DMSO. N = 3 biological replicates over 3 independent experiments. (**C**) Colony formation assay of *ZDHHC7* knockdown and control HCC cells with inhibitor or DMSO. N = 3 biological replicates over 3 independent experiments. (**D** to **F**) Mice were treated with MY-D-4 or vehicle and blood was collected. Body weight change (D), blood urea nitrogen (BUN), blood uric acid (BUA) (E), blood total bilirubin (TB), blood alanine transaminase (ALT) and blood aspartate transaminase (AST) (F) were evaluated. N = 5 mice for each group. Data represent the Mean ± SEM. ** p < 0.01, NS, not significant, by Two-tailed unpaired student’s t-test.

As a DHHC inhibitor, 2-BP (2-bromopalmitate) has several disadvantages. The IC50 (half maximal inhibitory concentration) for 2-BP is above 10 μM in vitro and it inhibits all reported ZDHHC gene expression without much selectivity(24, 25). Recently, a new DHHC inhibitor S-(2-acetamidoethyl) 2-bromohexadecanethioate (MY-D-4) with the IC50 around 1 μM for DHHC7 in vitro was reported(25). MY-D-4 is more potent than 2-BP in vitro and seems to be more potent at inhibiting DHHC7 than DHHC3/7, although its activity on other DHHCs were not tested. Compared to 2-BP, MY-D-4 has a stronger effect in inhibiting HCC cell proliferation (Fig. 5, A and B). Moreover, colony formation could not be further inhibited by MY-D-4 in the HCC cells with *ZDHHC7* depletion (Fig. 5C). We next evaluated the toxicity of MY-D-4 in vivo and found that intraperitoneal delivery of MY-D-4 had no signs of body weight loss (Fig. 5D) or organ dysfunction (Fig. 5, E and F).

**Fig. 6.**
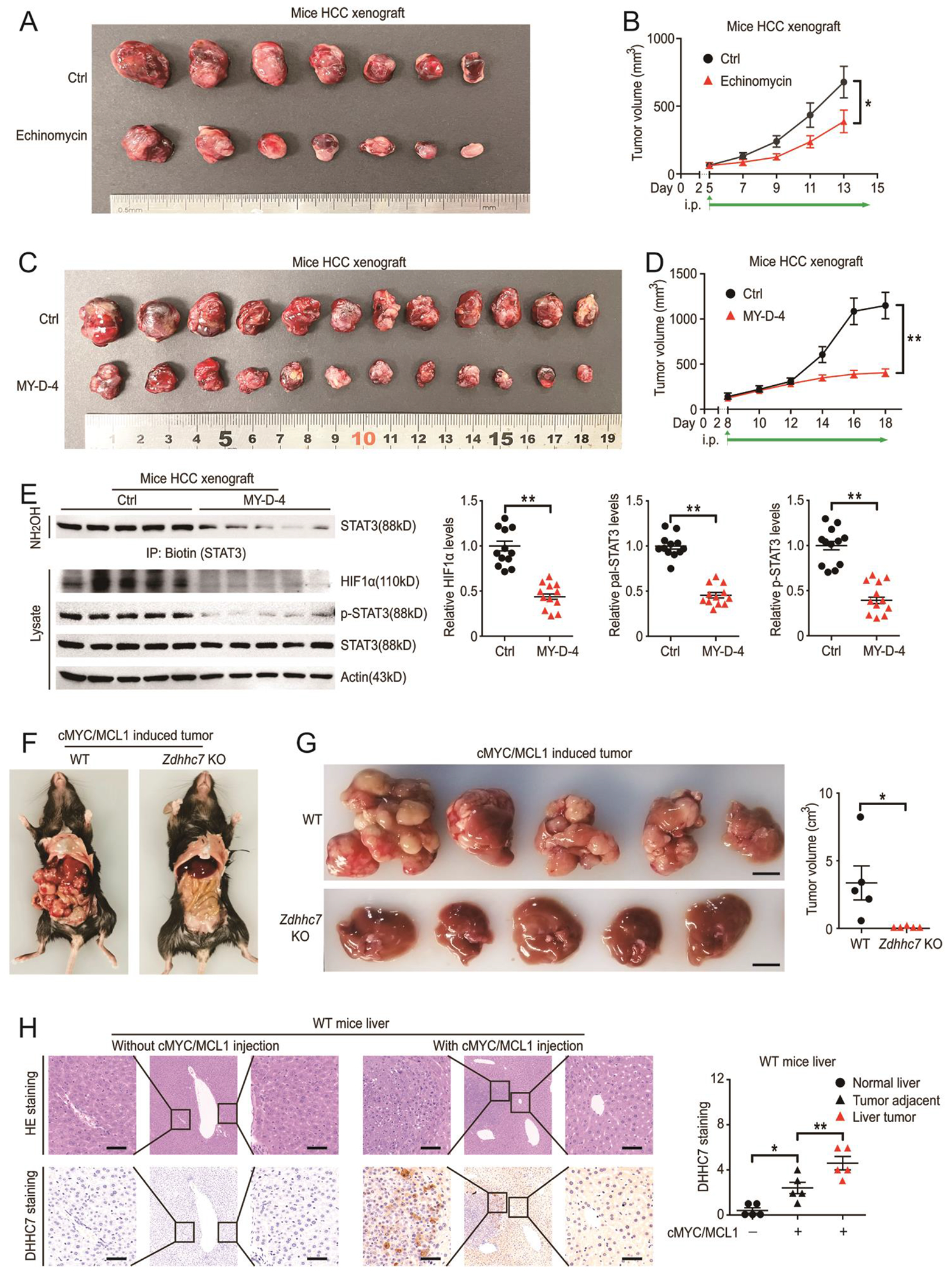
DHHC7 inhibition blocks HCC in vivo and leads to a decrease in STAT3 phosphorylation and palmitoylation. (**A** to **B**) The PLC/PRF/5 xenograft mouse model was used. Mice were treated with echinomycin or vehicle. Tumors were photographed (A) and tumor volumes were measured every 2 days quantified(B). N = 7 mice for each group. (**C** to **E**) The PLC/PRF/5 xenograft mouse model was used. Mice were treated with or without MY-D-4. Tumors were photographed (C) and tumor volumes were measured (D). Mice were sacrificed at the indicated time point and tumors analyzed by western blot (E). N = 12 mice for each group. (**F** to **G**) WT and *Zdhhc7* knockout mice were injected with c-Myc/MCL1 plasmids hydrodynamically through the tail vein. General morphology of mice (F), mouse liver morphology and quantification of tumor volumes (G) are shown. N = 5 mice for each group. (**H**) WT mice were injected with or without c-Myc/MCL1 plasmids hydrodynamically through the tail vein. DHHC7 staining and quantification of liver sections. N = 5 mice for each group. The scale bars in (G) are 1 cm. The scale bars of the in (H) are 50 μm. Data represent the Mean ± SEM. * p < 0.05, ** p < 0.01, by Two-tailed unpaired student’s t-test.

To evaluate whether the STAT3-HIF1α-DHHC7 loop could be a therapeutic target in vivo, we used the HIF1α inhibitor (echinomycin) and the DHHC7 inhibitor for further study, because inhibitors targeting on STAT3(26) and CDK5 (dinaciclib)(27, 28) had been proved effective in mice with HCC respectively. We found that echinomycin significantly inhibited the tumor growth compared to the control group in the xenograft mouse model with PLC/PRF/5 cells (Fig. 6, A and B). We further confirmed that MY-D-4 significantly inhibited the tumor growth compared to the control group in vivo (Fig. 6, C and D). This was associated with decreased S-palmitoylation and phosphorylation of STAT3 as well as HIF1α abundance in tumor lysates (Fig. 6E) which is consistent with in vitro assay (Fig 1).

To further validate whether DHHC7-induced STAT3-HIF1α-DHHC7 positive feedback loop contributes to the tumorigenesis of hepatic carcinoma cells. We applied a well-characterized murine HCC model induced by hydrodynamic co-expression of c-Myc and MCL1 oncogenes (c-Myc/MCL1) in the liver (9, 29). Noticeably, knockout of *Zdhhc7* significantly blunted tumorigenesis in HCC model (Fig. 6, F and G). Moreover, we confirmed that high DHHC7 could be induced in c-Myc/MCL1-treated mice liver compared to health control (Fig. 6H), suggesting that DHHC7 is involved in the malignancy of hepatic carcinoma cells and MY-D-4 is a promising inhibitor for HCC treatment.

### The upregulated STAT3, HIF1α and DHHC7 were confirmed in human HCC patients.

**Fig. 7.**
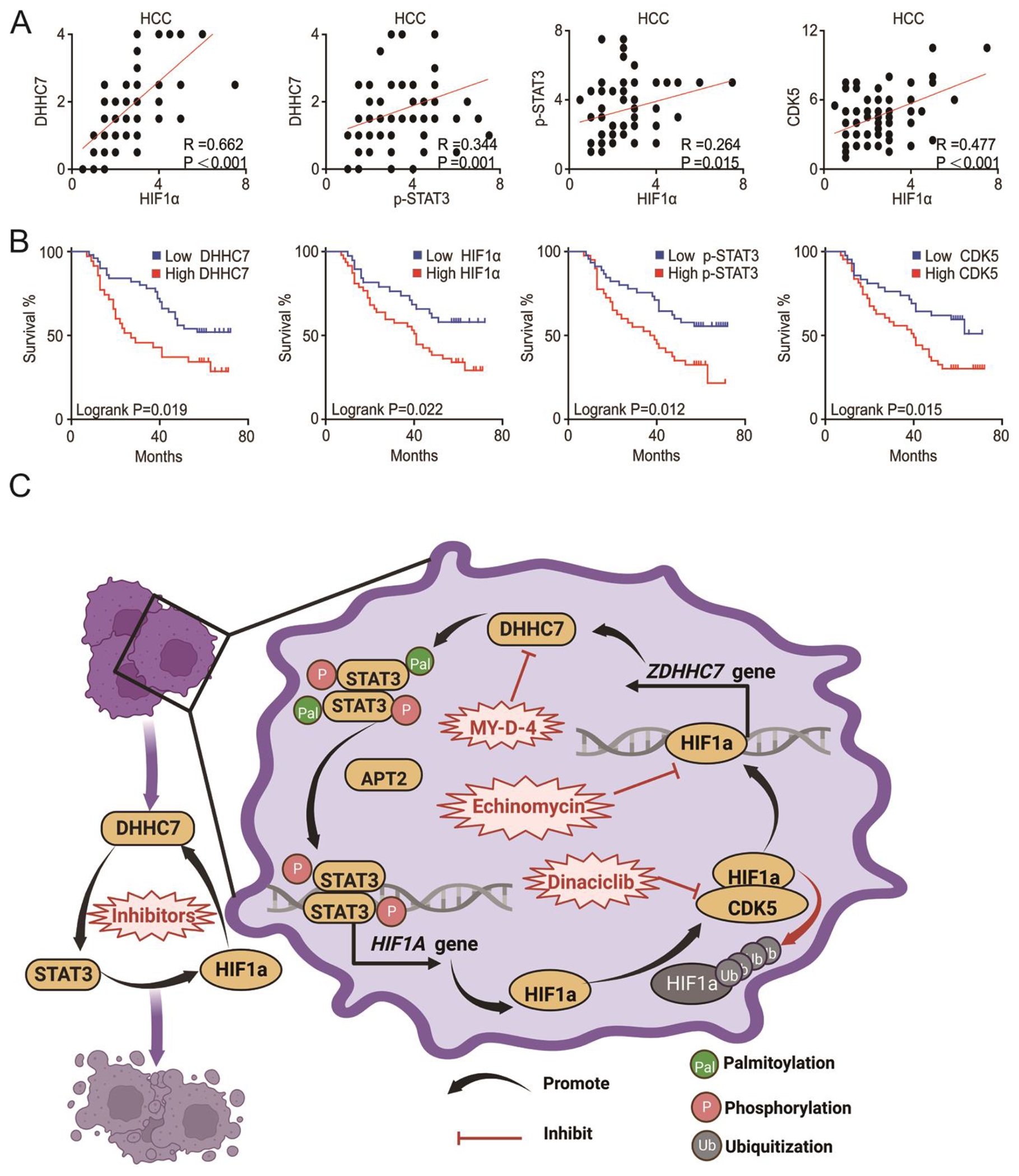
The DHHC7-STAT3-HIF1α positive feedback loop is upregulated in human HCC patients. (**A**) Human HCC tissues from 85 patients (Cohort 2) were analyzed using IHC. The correlation between DHHC7, HIF1α and p-STAT3 abundance were analyzed. N = 85 HCC patients. Indicated P value was calculated by Pearson correlation analysis. (**B**) Kaplan-Meier curves of DHHC7, HIF1α, p-STAT3 and CDK5 for HCC patients’ overall survival were performed, and the P value of each Kaplan-Meier curves were visualized. N = 85 HCC patients. (**C**) Working model of the DHHC7-STAT3-HIF1α positive feedback loop. In HCC cells, the palmitoyltransferase DHHC7 increased the transcriptional activity of STAT3 and the transcription of its target gene *HIF1A* by palmitoylating STAT3 on Cys108. Conversely, as a transcription factor, HIF1α directly promotes *ZDHHC7* gene expression. This creates a cycle between DHHC7 and HIF1α which is induced by STAT3 palmitoylation (left). The inhibition of DHHC7-HIF1α cycle blocks the malignancy of hepatic carcinoma cells (right).

Activated STAT3 and upregulated HIF1α promotes the malignancy of hepatic carcinoma cells and suggests a poor prognosis in HCC(3, 10, 17, 30–33). To determine whether the STAT3-HIF1α-DHHC7 loop is correlated with HCC in humans, another cohort HCC tissues from 85 patients were analyzed (Table S2) (Cohort 2). We found that protein abundance of DHHC7 and HIF1α exhibited a relatively high correlation with each other and p-STAT3 abundancemoderately correlated with both DHHC7 and HIF1α abundance in HCC tissues (Fig. 7A). Furthermore, patients with higher amounts of DHHC7 and HIF1α tended to have larger HCC tumors (fig. S4A). Higher amounts of DHHC7, HIF1α and p-STAT3 predicted poor prognosis in terms of overall survival and tumor free survival of HCC patients (Fig. 7B and fig. S4B and S4C). Collectively, the STAT3-HIF1α-DHHC7 loop leads to poor prognosis in HCC patients (Fig. 7C).

## Discussion

Although protein S-palmitoylation was discovered as a post-translational modification decades ago(34) and substantial progress has been made in elucidating the biological function of various palmitoyltransferases(12, 35), more work is needed regarding the regulation of DHHC and its involvement in HCC. Here we found that CDK5 and HIF1α can positively regulate DHHC7 expression and there is a positive feedback loop formed by DHHC7, STAT3, and HIF1α, which contributes to the malignancy of hepatic carcinoma.

Gene microarray combined TCGA data analysis is a beneficial approach to reveal the molecular mechanisms of diseases(20, 21). By examining all S-palmitoylation related enzymes with bioinformatics tools and cellular function study, we found that both *ZDHHC7* and *LYPLA2* were associated with the prognosis of HCC patients. Although it has been observed that the palmitoylation-depalmitoylation cycle induced by DHHC7 and APT2 is important for STAT3 activation cycles in Th17 cells(1), our data suggested that S-palmitoylation-related genes of STAT3 were positively correlated with *ZDHHC7* but not *LYPLA2* in HCC. Moreover, the amounts of STAT3 S-palmitoylation and phosphorylation were more sensitive to DHHC7 perturbation than APT2 perturbation. These data indicate that the regulation of STAT3 may depend on different cell types.

Our data identified that DHHC7 is a major contributor to the malignancy of HCC. TCGA data analysis was further confirmed by two different HCC cohorts and we found that DHHC7 was abundantly expressed in HCC and associated with tumor size and poor clinical prognosis. The palmitoyl acyltransferase DHHC7 has been indicated in nervous system disease(35, 36), glucose metabolism(37) and immune disease(1). Here, we showed mechanistically that DHHC7 palmitoylated and activated STAT3, which increased the expression *HIF1A*, which in turn promoted the transcription of *ZDHHC7*, forming a positive feedback loop and explaining why DHHC7 is a potential biomarker for HCC.

Due to the importance of DHHC7 in the positive feedback loop, DHHC7 could be the ideal target for the HCC treatment. Although, as a known palmitoyl acyltransferase inhibitor, 2-BP is reported to have anti-tumor effect(38), it is weak and non-specific (IC50 is around 16 μM for DHHC7 in vitro)(24, 25). A potential synthetic inhibitor, MY-D-4, based on the acylation-coupled lipophilic induction of the polarization (Acyl-cLIP) method, has an IC50 around 1 μM for DHHC7(25). Compared to 2-BP, MY-D-4 had a stronger effect on blocking HCC cell proliferation in vitro and our data suggests that MY-D-4 is a promising anti-tumor inhibitor with no obvious toxicity in vivo. Since *ZDHHC7* and *ZDHHC3/20* were genetically close to each other(19) and DHHC3/20 could also be inhibited by MY-D-4(25), we further evaluated whether other DHHCs involved in HCC cell proliferation. Unlike *ZDHHC7* silencing, no significant change of colony formation could be found with *ZDHHC3/20* silencing in HCC cells. Moreover, colony formation could not be further inhibited by MY-D-4 in HCC cells with *ZDHHC7* depletion. Collectively, DHHC7 is the primary target for MY-D-4 in proliferation blocking.

Constitutive activation of STAT3 contributes to the malignancy in many cancers including HCC and is associated with poor prognosis(3, 10). As a downstream target gene of STAT3, MCL1 artificially co-overexpressed with c-MYC in the liver induces in situ tumorigenesis in many different mouse strains and provides a perfect model for HCC research(9). This well-characterized murine HCC model was induced by hydrodynamical injection with c-Myc and MCL1 plasmids (c-Myc/MCL1). We found that knockout of *Zdhhc7* significantly blunted the c-Myc/MCL1 induced HCC, and this inhibition of tumorigenesis was associated with decreased S-palmitoylation and phosphorylation of STAT3 and decreased HIF1α abundance in tumors, consistent with our in vitro data. Collectively, this indicates that DHHC7 is involved in the malignancy of hepatic carcinoma cells and MY-D-4 is a promising inhibitor for HCC treatment.

We identified that the CDK5 as an important protein regulating the expression of ZDHHC7. HCC proliferation can be blocked by CDK5 inhibition(16, 17), and greater CDK5 expression is positively associated with the poor survival of HCC patients(16, 17). Our study showed that CDK5 could regulate the DHHC7-STAT3-HIF1α positive feedback loop by regulating HIF1α. Given that both STAT3 and HIF1α are important for various cancers and inflammatory diseases(1, 8, 39), the positive feedback loop constituted by DHHC7/STAT3/HIF1α can be a potential therapeutic target for treating many other malignant and inflammatory diseases.

## Materials and Methods

### Ethics approval.

The protocols for utilizing patients and cells were approved by the Ethical Committee of Drum Tower Hospital, Nanjing University. The protocols for mice study were approved by the Ethical Committee of Drum Tower Hospital, Nanjing University. This study was in compliance with all relevant ethical regulations. The baseline characteristics of cohort 1 and 2 are shown in Supplementary Information Table S1 and S2 respectively.

### Common reagents and antibodies.

The following reagents and antibodies were purchased from commercial sources: Inhibitor cocktail [Trichostatin A (TSA, T8552, Sigma, St Louis, MO, USA), protease inhibitor cocktail (P8340, Sigma), phosphatase inhibitor cocktail (P0044, Sigma)], Universal nuclease (88700, Thermo Fisher, Grand Island, NY, USA), Enzyme-linked chemiluminescence (ECL) plus (32132, Thermo Fisher), SYBR^®^ Green PCR Master Mix (4472908, Applied Biosystems, Grand Island, NY, USA), TB Green^®^ Premix Ex Taq^™^ II (RR820A, Takara, Kusatsu, Shiga, Japan). Streptavidin Agarose (20359, Thermo Fisher), Protein A/G PLUS-Agarose (sc-2003, Santa Cruz Biotechnology, Dallas, TX, USA), Anti-FLAG Agarose Gel (A2220, Sigma), Anti-HA Affinity Gel (E6779, Sigma), Dinaciclib (S2768, Selleck), Echinomycin (HY-106101, MCE), 2-BP (238422, Sigma). Antibodies were as follows: STAT3 (9139, CST, Danvers, MA, USA), Phospho-STAT3 (Tyr705) (ab76315, Abcam, Cambridge, UK), β-Actin (C4) HRP (SC-47778, Santa Cruz), Flag HRP (A8592, Millipore, Billerica, MA, USA), HA-probe (Y-11) (SC805, Santa Cruz), HA-probe (ab9110, Abcam), DHHC7 (ab254954, Abcam), DHHC7 (R12-3691, Assay Biotechnology, Fremont, CA, USA), HIF-1α (36169, CST), HIF-1α (ab51608, Abcam), CDK5 (2506, CST), Anti-mouse IgG HRP (7076S, CST), Anti-Rabbit IgG HRP (7074S, CST). S-(2-acetamidoethyl) 2-bromohexadecanethioate (MY-D-4) was synthesized according to the previously reported protocol(25).

### Cell culture and Plasmids.

Human HEK293T cells (obtained from ATCC) and human HepG2 and PLC/PRF/5 cells (obtained from Institute of Biochemistry and Cell Biology, Chinese Academy of Sciences) were grown in DMEM media (11965-092, Gibco, Carlsbad, NY, USA) with 10% bovine calf serum (CS, 12133C, Sigma). *CDK5* KO cells were generated with lentivirus encoding *CDK5* shRNA purchased from Genechem (Genechem, Shanghai, China). The sequence of *CDK5*-shRNA was #1, 5′-GGCCTTGAACACAGTTCCGT-3′. #2, 5′-ATCTCCCGGAGGGCGGAACT-3′. #3, 5′-CAGCGACAAGAAGCTGACTT-3′. Stable transfection of CDK5 knockdown lentivirus was conducted following manufacturer’s instructions. 2 μg/ml puromycin (P-600-100, GoldBio, St Louis, MO, USA) was added in culture media after transfection for 48 h and cells were seeded as a single cell in each well of 96-well plates using a limited dilution method. The knockout of CDK5 was confirmed by Western blot. The CDK5-Flag plasmids were purchased from Genechem (Genechem, Shanghai, China). The transient transfection was performed using FuGene 6 (E2691, Promega, Madison, WI, USA). APT2 and DHHC1-23 murine plasmids were kindly provided by Professor Masaki Fukata. STAT3 expression vectors with different tags were obtained from Addgene (Watertown, MA, USA). The HIF-1α plasmid and the pGL3-Basic-Firefly-luciferase plasmids were constructed by Generay Technologies (Generay, Shanghai, China). The pRL-TK-Renilla-luciferase plasmids were purchased from Promega (Madison, WI, USA). pT3-EF1a-c-MYC and pT3-EF1a-Mcl1 plasmids were kindly provided by Xin lab from University of California. Point mutations of plasmids were generated by QuikChange site-directed mutagenesis. *HIF1A* and *ZDHHC3/7/20* knockdown was performed by siRNA transfection using Dharma FECT 1 transfection reagent. The sequences of siRNA are listed as follows. *HIF1A* #1 sense: GUUGCCACUUCCACAUAAUTT, antisense: AUUAUGUGGAAGUGGCAACTT, #2 sense: CCGUAUGGAAGACAUUAAATT, antisense: UUUAAUGUCUUCCAUACGGTT, #3 sense: CAGGCCACAUUCACGUAUATT, antisense: UAUACGUGAAUGUGGCCUGTT. *ZDHHC3* #1 sense: GUGGGAACCAUGUGGUUUATT, antisense: UAAACCACAUGGUUCCCACTT, #2 sense: CGGGAAUAGAACAAUUGAATT, antisense: UUCAAUUGUUCUAUUCCCGTT, #3 sense: GCAUCAUCAACGGAAUUGUTT, antisense: ACAAUUCCGUUGAUGAUGCTT. *ZDHHC7* #1 sense: GCUCUUCACUAUGUAUAUATT, antisense: UAUAUACAUAGUGAAGAGCTT, #2 sense: GCUGCUGUAUUAAACCCGATT, antisense: UCGGGUUUAAUACAGCAGCTT, #3 sense: CAGUUAUGUUUGGCACCCATT, antisense: UGGGUGCCAAACAUAACUGTT. *ZDHHC20* #1 sense: CAGCCAAGAAAGACAACAATT, antisense: UUGUUGUCUUUCUUGGCUGTT, #2 sense: CAGCCUGUGACUCAUGUAUTT, antisense: AUACAUGAGUCACAGGCUGTT, #3 sense: CUCUCUUGGAUGCAGUAAATT, antisense: UUUACUGCAUCCAAGAGAGTT.

### Click chemistry and in-gel fluorescence detection.

Click chemistry assays were performed as described(1). Briefly, cells were treated with 50 μM palmitic acid analog Alkyne 14 (Alk14) for 5 hours and then collected and lysed in 1% NP-40 lysis buffer (25 mM Tris-HCl pH 8.0, 150 mM NaCl, 10% glycerol, 1% Nonidet P-40) with protease inhibitor cocktail. The target protein was purified with anti-flag agarose beads and click chemistry reagents were added to the beads in the following order: 1 μL of 4 mM TAMRA azide (47130, Lumiprobe, Hunt Valley, MD, USA), 1.2 μL of 10 mM Tris[(1-benzyl-1H-1,2,3-triazol-4-yl)methyl]amine, (TBTA) (T2993, Tcichemicals, Portland, OR, USA), 1 μL of 40 mM CuSO4, 1 μL of 40 mM Tris(2-Carboxyethyl)phosphine, HCl (TCEP, Hydrochloride) (580560, Millipore). The reaction mixtures were incubated for 30 mins in a dark at room temperature. SDS loading buffer was then added and the resulting mixture was heated at 95 °C for 10 min. Half of the mixture was also treated with hydroxylamine (438227, Sigma) (pH 7.4, final concentration 500 μM) and heated for another 5 min at 95 °C to remove S-palmitoylation. The samples were then resolved by SDS-PAGE. The gel was scanned to record the rhodamine fluorescence signal using a Typhoon 7000 Variable Mode Imager (GE Healthcare Life Sciences, Piscataway, NJ). After scanning, the gel was stained with Coomassie Brilliant Blue (CBB) (B7920, Sigma) to check for protein loading.

### Acyl-biotin exchange (ABE).

ABE assays were performed as described(1). Briefly, 50 U/ml nuclease (88700, Thermo Fisher) was added to samples and resulting mixture was suspended in 1 ml lysis buffer (100 mM Tris-HCl pH 7.2, 5 mM EDTA, 150 mM NaCl, 2.5% SDS, inhibitor cocktail) with 50 mM N-ethylmaleimide (NEM) (E3876, Sigma). Samples were solubilized and centrifuged at 16,000 g for 20 min. 2 μg protein for each sample was precipitated with chloroform/methanol/water (volume ratio 1:4:3), briefly air-dried, and dissolved in 1 ml of lysis buffer with 5 mM Biotin-HPDP (16459, Cayman Chemical, Ann Arbor, MI, USA) by gentle mixing at RT. Samples were divided equally and incubated with 0.5 ml of 1 M hydroxylamine or 1M NaCl respectively at RT for 3 hours. Samples were precipitated again and dissolved in 200 μl of resuspension buffer [100 mM Tris-HCl pH 7.2, 2% SDS, 8 M urea, 5 mM EDTA]. For each sample, 20 μl was used as loading control and 180 μl was diluted 1:10 with PBS and incubated with 20 μl of streptavidin beads with shaking overnight at 4°C. Beads were washed 3 times with PBS containing 1% SDS. The beads and loading controls were mixed with SDS loading buffer and heated at 95 °C for 10 min. Samples were subjected to western blot analyses.

### DEGs of paired-HCC from TCGA data.

The HCC RNA-Seq data were downloaded from the TCGA database using The GDC Data Portal (https://gdc-portal.nci.nih.gov/). Differentially expressed genes in HCC and normal counterpart samples were visualized by Gepia (http://gepia.cancer-pku.cn/). Kaplan-Meier curves of all genes for HCC patients’ overall survival were performed in Gepia. The sequencing data were all publicly available and no ethical issues were involved.

### Gene identification with GENEVESTIGATOR.

Genevestigator (https://www.genevestigator.com/gv/) is a well-annotated database of microarray experiments and powerful tool search engine for gene expression with clustering analysis^21,22^. Three different microarray experiments (HS-01450(LINCS), HS-02631(GSE99340) and HS-00243(GSE7835)) tagged with the term ‘ZDHHC7’ have included here after exclusion of irrelevant matches. Genes that evidenced a low standard deviation and appropriate expression value on both chips were used for analysis. To find conditions relevant for the gene of interest, perturbations tool was applied. Furthermore, co-expression tool was used to find the most likely co-expressed genes with the *ZDHHC7* in all available liver cancer tissues and cell lines.

### Transcription factor binding analysis.

We scanned for candidate transcription factor binding motifs of *ZDHHC7* using JASPAR database (http://jaspar.genereg.net/): For these scans, we used DNA regions of 2000 bp upstream (− 2000) and 100 bp downstream (+ 100) as search windows relative to the binding locations. The relative score is a threshold score in the range 0 to 1; a higher score correlates to a greater likelihood of binding.

### Luciferase Reporter Assays.

Cells were seeded in 96-well plate and transfected with indicated plasmids. After 24 h, the cells were lysed, and luciferase activity was determined using the Dual-Luciferase Reporter Assay system (E2920, Promega). In hypoxic groups, cells were switched to hypoxia culture condition (1% O_2_) for 4 h, followed by lysis and luciferase activity measurement.

### Mouse models.

To establish subcutaneous xenograft tumor models, 4-6-week-old male Balb/c nude mice were quarantined for 1 week before inoculation of PLC/PRF/5 cells. Stable PLC/PRF/5 cells (1×10^6^) were suspended in 100 μl PBS and injected subcutaneously into the flanks of the mice. Mice were injected with M-Y-D4 at 30mg/kg body weight intraperitoneally every 2 days as indicated. The MY-D-4 powder was dissolved in 150 μl olive oil (A502795, Sangon Biotech, Shanghai, China) before injection and the tumor volumes were measured every 2 days. Tumor volume was calculated with the formula A×B^2^/2 (A-length, B-width). The mice were sacrificed at the indicated time point and tumors were harvested for subsequent analysis. To establish the c-Myc/MCL1 induced tumorigenesis mouse models, B6.129P2(FVB)-Zdhhc7tm1.2Lusc/Mmmh, RRID:MMRRC_043511-MU mice were obtained from the Mutant Mouse Resource and Research Center (MMRRC) at University of Missouri. Genotype identification was performed according to the MMRRC protocol. Primers for the WT allele were as follows: forward: TGAGCCAGGATGGATTTCAGACA and backward: TGCCCTCGGACGCAGGAGATGAA. Primers for the mutant type allele were as follows: forward: TCCCCTGATGTATGCGAATGTCC and backward: AACAGGTGCCTTTTGAATGTCAG. 7-week-old male WT and *Zdhhc7* knockout mice were treated as previously indicated (9, 29). In brief, 12 mg of pT3-EF1a-c-MYC with 6 mg of pT3-EF1a-Mcl1 along with 1.5 μg sleeping beauty transposase were diluted in 2 mL of saline (0.9% NaCl), filtered through 0.22-μm filter, and injected into the lateral tail vein of mice in 5 seconds. Mice were housed, fed, monitored and sacrificed when moribund. In terms of mice allocation, comparable mice were randomly assigned into different groups. The number of the animals is based on previous research and our experience.

### rtPCR.

For the gene expression analysis, the rtPCR was performed using SYBR^®^ Green PCR Master Mix according to the manufacturer’s standard protocol. Primer sequences were from references(8, 31, 40). The cDNA microarray kits were commercially obtained (HLivH060PG02, Outdo Biotech, Shanghai, China) and the clinical characteristics of 30 patients with HCC (Cohort 1) was summarized in Table S1. rtPCR primer sequences are listed as follows, *ZDHHC7* forward: TGCAGACTTCGTGGTGACTTTCG, *ZDHHC7* reverse: TGGGGCACTTGTAGATGACTTCC, *HIF1A* forward: CTCAAAGTCGGACAGCCTCA, *HIF1A* reverse: CCCTGCAGTAGGTTTCTGCT.

### Immunohistochemistry.

The tissue microarray slides were commercially obtained (HLivH180Su09, Outdo Biotech, Shanghai, China) and the clinical characteristics of 85 patients with HCC (Cohort 2) was summarized in Table S2. Mice samples staining was performed by Servicebio (Servicebio, Wuhan, China). Sections were treated with immunoperoxidase using the DAB kit (ZLI-9017, Zsbio, Beijing, China) and then scored. Staining intensity was graded as follows: absent staining = 0, weak = 1, moderate = 2, and strong = 3. The percentage of staining was graded as follows: 0 (no positive cells), 1 (<25% positive cells), 2 (25% - 50% positive cells), 3 (50% - 75% positive cells), and 4 (>75% positive cells). The score for each tissue was calculated with multiplication by two pathologists respectively, and the range of this calculation was therefore 0 - 12.

### Colony formation assay.

Different HCC cells (1000 cells/well) were seeded in six-well plates and treated with or without DHHC7 inhibitor (50 μM) for 48 h respectively, then inhibitors were removed and cells were cultured for another 10 to14 days until visible colonies were formed. The colonies were fixed with formaldehyde and stained with crystal violet and counted.

### Statistical Analysis.

Quantitative analyses were performed with SPSS 17.0 and data was expressed as means ± standard error (SE). Comparisons among groups were performed by Student’s t-test and other data were analyzed using a one-way analysis of variance (ANOVA).

## Supplementary Material

1

## Data Availability

All data needed to evaluate the conclusions in the paper are present in the paper or the Supplementary Materials Data are available from the corresponding author upon request.
