# Supplementary material for: STAT3 palmitoylation initiates a positive feedback loop that promotes the malignancy of hepatocellular carcinoma cells in mice
Source: Sci Signal. Author manuscript; Available in PMC 2024 Mar 2. (PMC10907978; doi:10.1126/scisignal.add2282)
Supplement: 1 [file NIHMS1968456-supplement-1.pdf]

Supplementary Materials: Figs. S1-S4 & Tables S1-S2

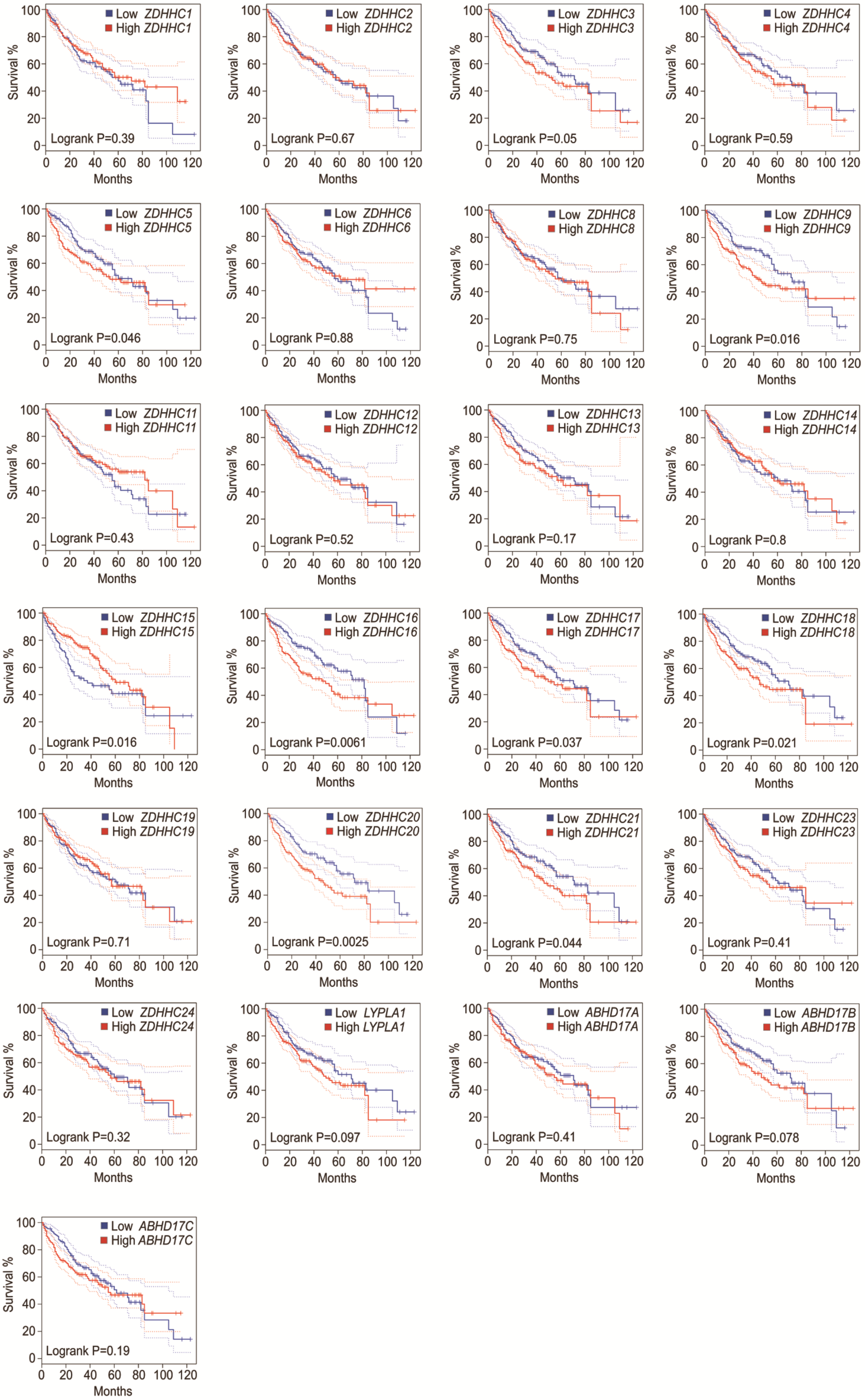

Supplementary Fig. 1. Kaplan-Meier curves of genes encoded palmitoyltransferases and acyl

protein thioesterases family. The expression levels of palmitoyltransferases (DHHCs) and acyl protein thioesterases (APT and ABHD family members) genes in HCC (N = 369) samples from TCGA data were visualized using Gepia (http://gepia.cancer-pku.cn/). Kaplan-Meier curves of genes were performed in Gepia and visualized respectively.

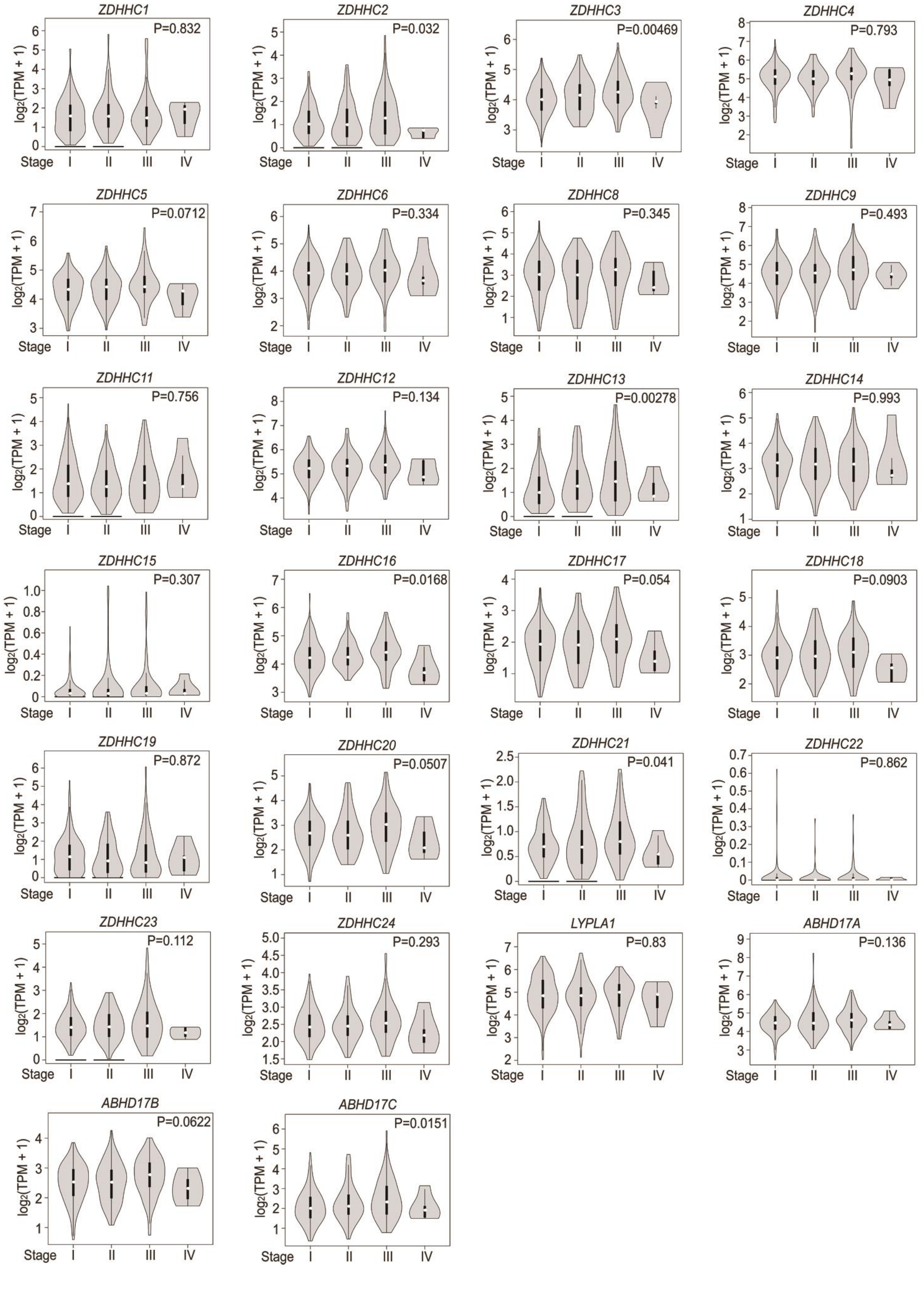

Supplementary Fig. 2. Expression levels of genes encoded palmitoyltransferases and acyl protein

thioesterases family in different stages of HCC patients. (**A**) The expression levels of palmitoyltransferases (DHHCs) and acyl protein thioesterases (APT and ABHD family members) genes in HCC (N = 369) samples from TCGA data were visualized using Gepia (http://gepia.cancer-pku.cn/). The expression levels of genes in different stages of HCC patients were performed in Gepia and visualized respectively.

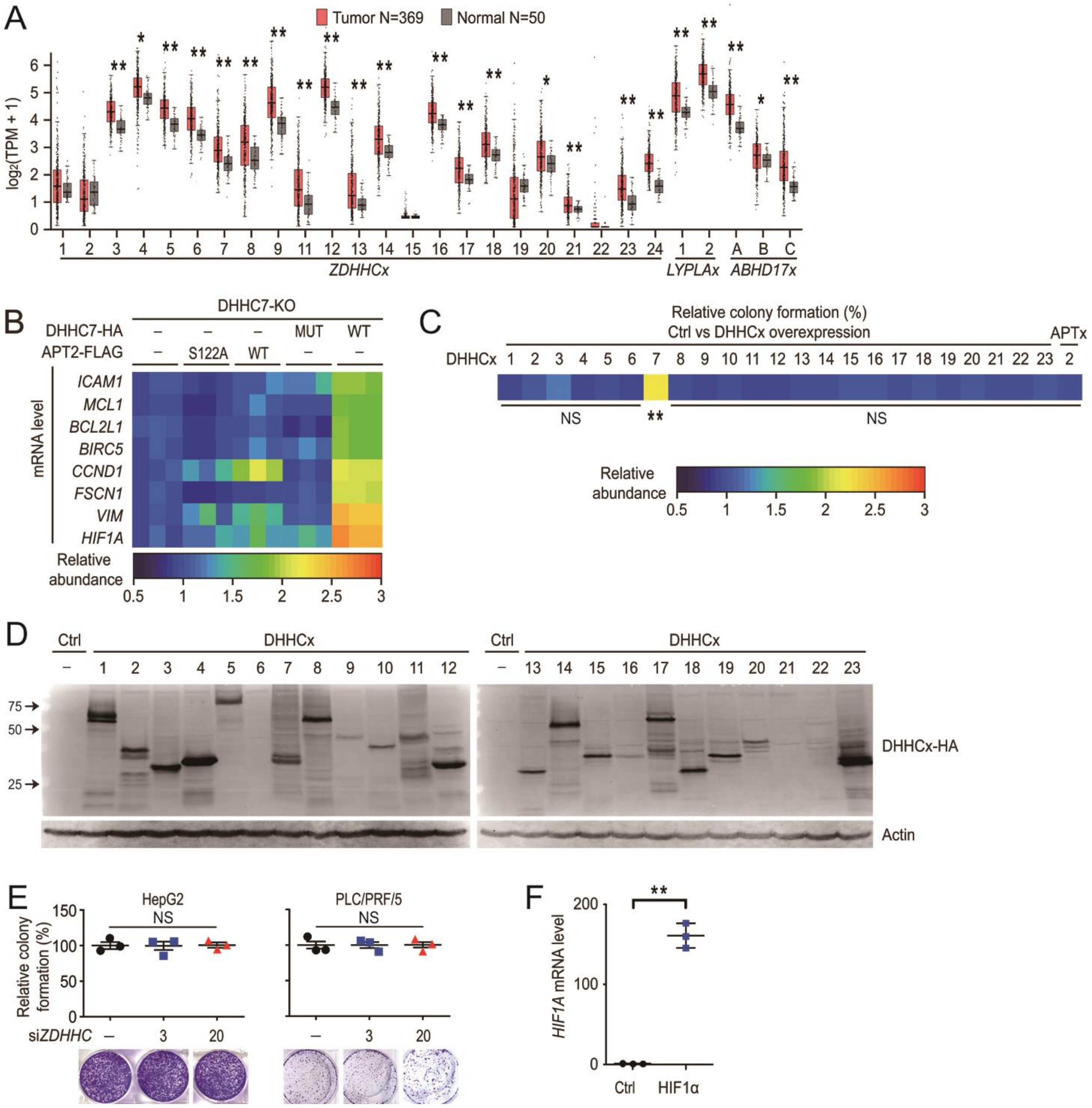

Supplementary Fig. 3. DHHC7 is most relevant to malignancy of HCC among palmitoyltransferases and acyl protein thioesterases. (**A**) Differentially expressed genes in HCC (N = 369) and normal liver (N = 50) samples from TCGA data were visualized using Gepia (http://gepia.cancer-pku.cn/) and visualized respectively. TPM, Transcripts Per Million. (**B**) DHHC7 KO 293T cells were transfected with different HA-DHHC7 and Flag-APT2 plasmids as indicated. The total mRNA was extracted and rtPCR analysis of the indicated Mrna is shown. The relative change compared to the control counterpart was visualized. N = 3 biological replicates over 3 independent experiments. (**C**) HepG2 cells were transfected with Flag-APT2 or HA-DHHCs plasmids. P values and relative increase of cell colony formation from indicated targets compared to

the control were visualized. N = 3 biological replicates over 3 independent experiments. (**D**) The expression of DHHCs were visualized by western blot. N = 3 biological replicates over 3 independent experiments. (**E**) *ZDHHC3/20* knockdown HCC cells and control counterparts were seeded in plates for culture and colony numbers in each well of the plate were counted and normalized as indicated. N = 3 biological replicates over 3 independent experiments. (**F**) The overexpression of HIF1α were visualized by rtPCR. N = 3 biological replicates over 3 independent experiments. Data represent the Mean ± SEM. * $p < 0.05$, ** $p < 0.01$, NS not significant, by Two-tailed unpaired student's t-test.

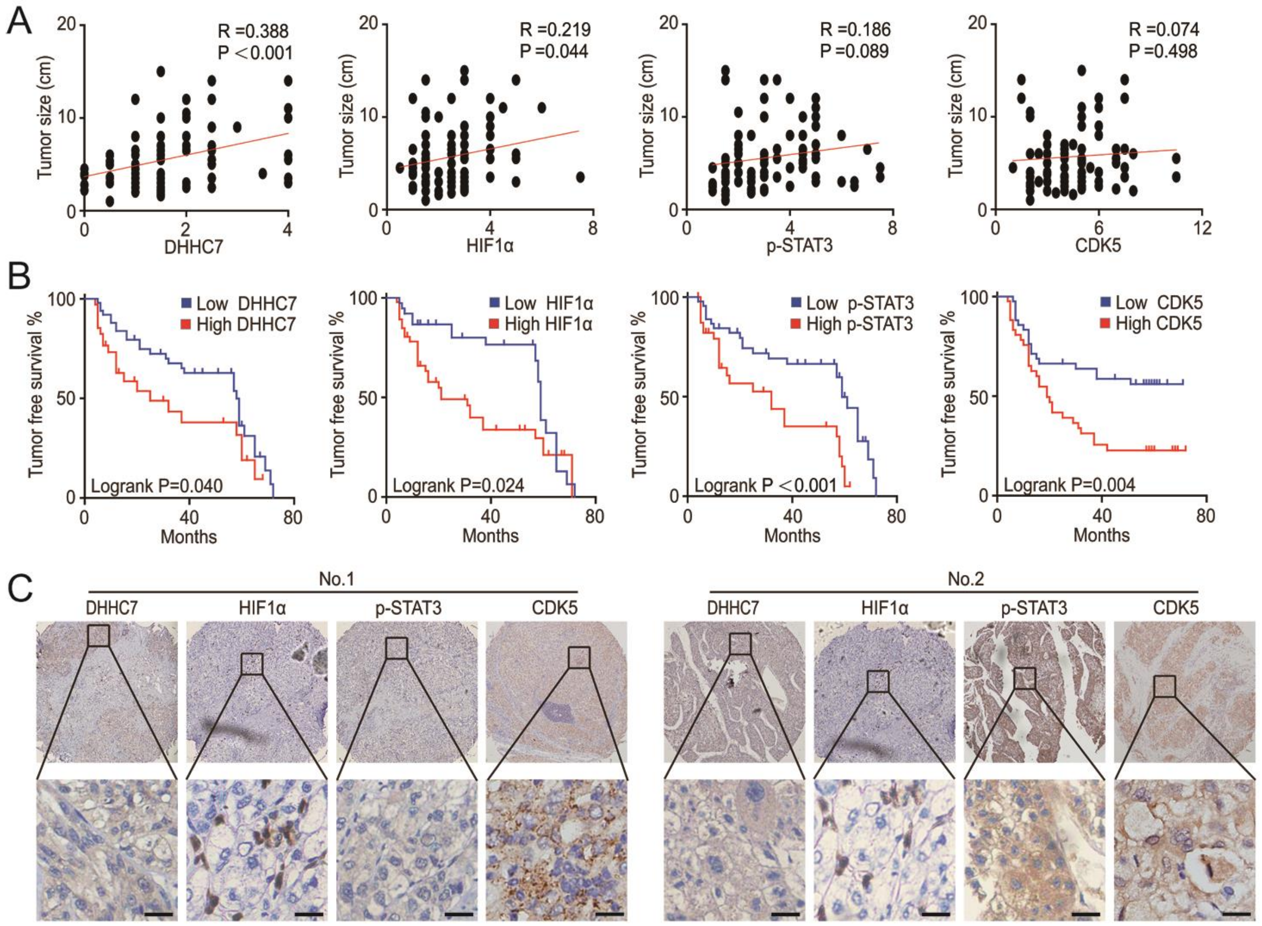

Supplementary Fig. 4. The DHHC7-STAT3-HIF1α positive feedback loop promotes the malignancy of HCC. (**A** to **C**) Human HCC tissues from 85 patients (Cohort 2) were analyzed using IHC. N = 85 HCC patients. (A) The correlation between tumor size and targets were analyzed. Indicated P value was caculated by Pearson correlation analysis. (B) Kaplan-Meier curves of DHHC7, HIF1α, p-STAT3 and CDK5 for HCC patients' tumor free survival were performed, and the P value of each Kaplan-Meier curves were visualized. (C) IHC staining for the targets were visualized. Scale bars, 50 μm.

Tables and legends

Table S1: Clinical characteristics of 30 patients with HCC (Cohort 1)

		DHHC7 expression, n (%)			
	n	Low (%)	High (%)	X^2	P value
Gender				2.516	0.193
Male	23	11(47.8)	12(52.2)		
Female	7	1(14.3)	6(85.7)		
Age				1.172	0.442
≤60	19	9(47.4)	10(52.6)		
＞60	11	3(27.3)	8(72.7)		
Size (mm)				8.167	0.008
＜50	13	9(69.2)	4(30.8)		
≥50	17	3(17.6)	14(82.4)		
Vascular involvement				0.089	1.000
Yes	14	6(42.9)	8(57.1)		
No	16	6(37.5)	10(62.5)		
AJCC Stage				0.089	1.000
1	0	0	0		
2	14	6(42.9)	8(57.1)		
3-4	16	6(37.5)	10(62.5)		
Cirrhosis				0.362	0.711
Positive	13	6(46.2)	7(53.8)		
Negative	17	6(35.3)	11(64.7)		
HBsAg				0	1.000
Positive	15	6(40.0)	9(60.0)		
Negative	15	6(40.0)	9(60.0)		

Table S2: Clinical characteristics of 85 patients with HCC (Cohort 2)

	n	DHHC7 expression, n (%)				HIF1α expression, n (%)				p-STAT3 expression, n (%)				CDK5 expression, n(%)			
		Low (%)	High (%)	X^2	P value	Low (%)	High (%)	X^2	P value	Low (%)	High (%)	X^2	P value	Low (%)	High (%)	X^2	P value
Gender				3.372	0.086			3.554	0.086			3.040	0.095			1.884	0.170
Male	70	38 (54.3)	32 (45.7)			28 (54.3)	42 (45.7)			34 (54.3)	36 (45.7)			37(52.9)	33(47.1)		
Female	15	12 (80.0)	3 (20.0)			10 (80.0)	5 (20.0)			11 (80.0)	4 (20.0)			5(33.3)	10(66.6)		
Age				2.732	0.109			0.107	0.791			0.295	0.787			0.106	0.745
≤60	68	43 (63.2)	25 (36.8)			31 (63.2)	37 (36.8)			35 (63.2)	33 (36.8)			33(48.5)	35(51.5)		
＞60	17	7 (41.2)	10 (58.8)			7 (41.2)	10 (58.8)			10 (41.2)	7 (58.8)			9(52.9)	8(47.1)		
Size (mm)				13.989	＜0.001			3.239	0.084			8.825	0.004			0.944	0.331
＜50	40	32 (80.0)	8 (20.0)			22 (80.0)	18 (20.0)			28 (80.0)	12 (20.0)			22(55.0)	18(45.0)		
≥50	45	18 (40.0)	27 (60.0)			16 (40.0)	29 (60.0)			17 (40.0)	28 (60.0)			20(44.4)	25(55.6)		
Differentiation				2.346	0.310			1.995	0.369			3.941	0.139			2.318	0.314
Well	2	1 (50.0)	1 (50.0)			1 (50.0)	1 (50.0)			0 (50.0)	2 (50.0)			0(0.0)	2(100.0)		
Medium	61	39 (63.9)	22 (36.1)			30 (63.9)	31 (36.1)			31 (63.9)	30 (36.1)			32(52.5)	29(47.5)		
Poor	22	10 (45.5)	12 (54.5)			7 (45.5)	15 (54.5)			14 (45.5)	7 (54.5)			10(45.5)	12(54.5)		
AJCC Stage				5.530	0.022			0.817	0.491			1.163	0.36			2.321	0.128

1	56	38(67.9)	18(32.1)			27(67.9)	29(32.1)			32(67.9)	24(32.1)			31(55.4)	25(44.6)		
2	29	12(41.4)	17(58.6)			11(41.4)	18(58.6)			13(41.4)	16(58.6)			11(37.9)	18(62.1)		
3-4	0																
Cirrhosis				0.97	1.000			2.195	0.211			0.163	0.762			0.002	0.965
Negative	12	7(58.3)	5(41.7)			3(58.3)	9(41.7)			7(58.3)	5(41.7)			6(50.0)	6(50.0)		
Positive	73	43(58.9)	30(41.1)			35(58.9)	38(41.1)			38(58.9)	35(41.1)			36(49.3)	37(50.7)		
HBsAg				1.017	0.367			0.519	0.551			1.635	0.240			0.121	0.728
Negative	13	6(46.2)	7(53.8)			7(46.2)	6(53.8)			9(46.2)	4(53.8)			7(53.8)	6(46.2)		
Positive	72	44(61.1)	28(38.9)			31(61.1)	41(38.9)			36(61.1)	36(38.9)			35(48.6)	37(51.4)		
AFP				0.006	1.000			0.098	0.824			29.253	＜0.001			1.585	0.208
＜400	53	31(58.5)	22(41.5)			23(58.5)	30(41.5)			16(58.5)	37(41.5)			29(54.7)	24(45.3)		
≥400	32	19(59.4)	13(40.6)			15(59.4)	17(40.6)			29(59.4)	3(40.6)			13(40.6)	19(59.4)		
